# Cardiovascular disease and all-cause mortality associated with individual and combined cardiometabolic risk factors

**DOI:** 10.1186/s12889-023-16659-8

**Published:** 2023-09-05

**Authors:** Xue Cao, Linfeng Zhang, Xin Wang, Zuo Chen, Congyi Zheng, Lu Chen, Haoqi Zhou, Jiayin Cai, Zhen Hu, Yixin Tian, Runqing Gu, Yilin Huang, Zengwu Wang

**Affiliations:** Division of Prevention and Community Health, National Center for Cardiovascular Disease, National Clinical Research Center of Cardiovascular Disease, State Key Laboratory of Cardiovascular Disease, Fuwai Hospital, Peking Union Medical College & Chinese Academy of Medical Sciences, No. 15 (Lin), Fengcunxili, Mentougou District, Beijing, 102308 China

**Keywords:** Cardiometabolic risk factor, Cardiovascular disease, Population attributable fraction

## Abstract

**Background:**

Previous studies have investigated the association between cardiometabolic risk factors and cardiovascular disease (CVD), but evidence of the attributable burden of individual and combined cardiometabolic risk factors for CVD and mortality is limited. We aimed to investigate and quantify the associations and population attributable fraction (PAF) of cardiometabolic risk factors on CVD and all-cause mortality, and calculate the loss of CVD-free years and years of life lost in relation to the presence of cardiometabolic risk factors.

**Methods:**

Twenty-two thousand five hundred ninety-six participants aged ≥ 35 without CVD at baseline were included between October 2012 and December 2015. The outcomes were the composite of fatal and nonfatal CVD events and all-cause mortality, which were followed up in 2018 and 2019 and ascertained by hospital records and death certificates. Cox regression was applied to evaluate the association of individual and combined cardiometabolic risk factors (including hypertension, diabetes and high low-density lipoprotein cholesterol (LDL-C)) with CVD risk and all-cause mortality. We also described the PAF for CVD and reductions in CVD-free years and life expectancy associated with different combination of cardiometabolic conditions.

**Results:**

During the 4.92 years of follow-up, we detected 991 CVD events and 1126 deaths. Hazard ratio were 1.59 (95% confidential interval (CI) 1.37–1.85), 1.82 (95%CI 1.49–2.24) and 2.97 (95%CI 1.85–4.75) for CVD and 1.38 (95%CI 1.20–1.58), 1.66 (95%CI 1.37–2.02) and 2.97 (95%CI 1.88–4.69) for all-cause mortality, respectively, in participants with one, two or three cardiometabolic risk factors compared with participants without diabetes, hypertension, and high LDL-C. 21.48% of CVD and 15.38% of all-cause mortality were attributable to the combined effect of diabetes and hypertension. Participants aged between 40 and 60 years old, with three cardiometabolic disorders, had approximately 4.3-year reductions life expectancy compared with participants without any abnormalities of cardiometabolic disorders.

**Conclusions:**

Cardiometabolic risk factors were associated with a multiplicative risk of CVD incidence and all-cause mortality, highlighting the importance of comprehensive management for hypertension, diabetes and dyslipidemia in the prevention of CVD.

**Supplementary Information:**

The online version contains supplementary material available at 10.1186/s12889-023-16659-8.

## Introduction

Cardiovascular disease (CVD) remains the leading cause of death in China, and nearly 4.58 million deaths were from CVD in 2020 [1]. Over the past two decades, China has experienced rapid economic and demographic transitions, CVD burden are likely to increase due to the aging population and unfavorable life-style behaviors that can lead to an increase in hypertension, dyslipidemia, diabetes as well as other non-communicable disorders. It has been estimated that the prevalence of hypertension, diabetes and dyslipidemia in China were 23.2%, 11.2% and 33.8% [2–4], respectively, which may contribute a larger CVD burden to society and government, because hypertension, diabetes, and hyperlipidemia are the major metabolically attributable risk factors for CVD disease burden [5, 6]. Moreover, hypertension, high cholesterol, and hyperglycemia often coexist, which have common metabolic abnormalities, and real-world evidence found that approximately 66% of those diagnosed with diabetes had dyslipidemia and hypertension [7].

Previous studies have investigated the association of blood pressure, blood glucose, and lipids with CVD and all-cause mortality risk and their attributable disease burden separately [8, 9]. However, few have systematically clarified the association between combined cardiometabolic disorders and CVD and all-cause mortality, especially for Chinese people. To our best knowledge, only one study has investigated the associations of individual and combined hypertension, diabetes and dyslipidemia with CVD risk among Chinese, and found that both individual and combined cardiometabolic risk factors are related to CVD risk, and these three risk factors showed a multiplicative risk of CVD incidence. However, this study had a relatively short follow-up duration, which might limit the statistical power [10]. Besides, the evidence regarding the attributable burden of comorbidities for CVD and mortality was insufficient, and the relatively few data from China were involved. Beyond that, few studies have quantitatively assessed such relationships using absolute metrics such as disease-free years, which was easy to understand for both the general public and healthcare professionals [11, 12].

Therefore, it is of paramount importance to clarify and quantify both the individual and combined associations and PAF of cardiometabolic risk factors, including hypertension, diabetes and high LDL-C, with the risk of CVD and mortality, and calculate the loss of CVD-free years and years of life lost in relation to the presence of cardiometabolic risk factors.

## Materials and methods

### Study population

China Hypertension Survey (CHS) is a nationally representative population-based study, which recruiting ~ 0.5 million participants from 31 provinces between October 2012 to December 2015 in mainland China. Design details were published previously [13–15]. This sub-study was based on CHS, and 16 cities and 17 counties were selected using a simple random sampling method from eastern, central, and western regions according to their geographical location and economic level [16]. Next, at least three communities or villages were randomly selected from each region. Then a given number of participants aged ≥ 35 years were selected from communities or villages. Finally, a total of 30,036 participants with incomplete physical examinations at baseline between 2012 to 2015 were followed up in 2018–2019. This study was approved by the Ethics Committee of Fuwai Hospital (Beijing, China) and performed under the guidelines of the Helsinki Declaration. Informed consent was obtained from each participant.

We dropped out participants who lost to follow-up (n = 4711), then subjects with medical history of CVD (coronary heart disease (CHD), stroke and other heart diseases that are not clearly defined, *n* = 2135) and 594 (2.0%) participants with missing data on covariates at baseline were also excluded. Ultimately, we included 22,596 participants who were free of CVD and had complete information on diabetes, hypertension, and lipids at baseline into the main analysis (Figure S1 in supplementary materials).

### Definition and classification of cardiometabolic factors

Prevalent hypertension was defined as systolic blood pressure (SBP) ≥ 140 mmHg, and /or diastolic blood pressure (DBP) ≥ 90 mmHg, and/or self-reported physician-diagnosed hypertension, and/or medication use for antihypertensive at baseline. According to Chinese Diabetes Society 2017 criteria, diabetes was defined as fasting plasma glucose (FPG) level of 7.0 mmol/L (126 mg/dL) or more, or self-reported previous diagnosis for type 2 diabetes mellites by health care professionals, or taking anti-diabetic drugs [17]. Low-density lipoprotein cholesterol (LDL-C) was selected as the primary lipid factor of interest, and LDL-C ≥ 4.12 mmol/L (160mg/dL) was considered as high LDL-C [18].

### Covariates

At baseline, information on demographic characteristics, lifestyle risk factors, and medical history were collected by well-trained interviewers via a structured questionnaire. Age of participants was categorized into five groups: 35–44, 45–54, 55–64, 65–74, and ≥ 75 years old. Educational attainment was classed into two groups: middle-high school or lower and high school or above. Information on employment status was also collected through self-reporting and the classification included employed, retired, students and unemployed. Participants who had at least one parent or siblings with a medical history of CVD was considered as having family history of CVD. Health-related factors included alcohol consumption, smoking (never, former and current) and medical history of CVD (CHD, stroke and other heart diseases that are not clearly defined). In the past month, participants who had a history of drinking at least once per week were defined as alcohol consumption. Height and body weight were measured with participants wearing thin clothing using a standardized right-angle device and an OMRON body fat and weight measurement device (V-body HBF-371, Omron, Japan). Body mass index (BMI), computed by dividing weight (kg) by height squared (m^2^), was divided into < 18.50 kg/m^2^, 18.50–23.9 kg/m^2^, 24.00–27.9 kg/m^2^, and ≥ 28 kg/m^2^ [19]. Resting Blood pressure was measured 3 times on the right arm in the sitting position after resting at least for 5 min using the Omron HBP-1300 professional portable blood pressure monitor (Omron, Kyoto, Japan), with 30 s between each measurement. The average of the three readings was used for the final analysis. Laboratory tests including TC, TG, HDL-C, LDL-C and FPG were detected by a central core laboratory (Beijing Adicon Clinical Laboratories, Inc., Beijing, China) by collecting venous blood for at least 8 h fasting.

### Ascertainment of incident CVD events and mortality

The outcomes were the composite of fatal and nonfatal CVD events and mortality, and CVD events were defined as combined CHD, stroke, chronic heart failure, and death due to CVD. CHD was defined as non-fatal CHD (including myocardial infarction, coronary artery bypass graft surgery, or percutaneous coronary intervention) and fatal CHD (such as fatal myocardial infarction and other coronary deaths). Stroke included non-fatal stroke and fatal stroke (subarachnoid hemorrhage, intracerebral hemorrhage, ischemic stroke, unspecified stroke). Mortality outcomes included all-cause mortality and cause-specific mortality. CVD events and deaths were initially identified by trained health care staff, and then ascertained by the central adjudication committee of Fuwai Hospital (Beijing, China) through verification of hospital records and death certificates. Specifically, for those who have been hospitalized due to CVD, CVD events were evaluated based on the medical records or diagnosis and treatment records, such as medical history, main symptoms and signs and medical examinations, etc.; or for emergency patients, we verified these events via medical history, the disease process, the local doctor's diagnosis and treatment provided by their relatives and local doctors. All events were coded according to the International Classification of Diseases, 10th Revision (ICD-10) by trained healthcare staffs blinded to baseline information. Moreover, during the follow-up, we conducted strict quality control by formulating a unified work plan and rigorous training, and the incident Diagnosis Committee has successively evaluated the completeness and accuracy of case identification. Additionally, we randomly selected 10% of the participants to check the false negative rate.

### Statistical analysis

In this present study, the status of cardiometabolic disorder were categorized into the one of diabetes, hypertension, and high LDL-C and those with two or three combined cardiometabolic disorders at the same time according to the numbers of cardiometabolic disease at baseline. Besides, we also categorized subjects in to the following 8 mutually exclusive groups according to baseline the status of cardiometabolic disease: (1) none of these (reference group), (2) diabetes, (3) high LDL-C, (4) hypertension, (5) diabetes and high LDL-C, (6) diabetes and hypertension, (7) high LDL-C and hypertension, and (8) diabetes, high LDL-C, and hypertension.

Data were described as means (standard deviation) for continuous variables and frequency and percentages for categorical variables according to the numbers of cardiometabolic disorders at baseline. The Kolmogorov–Smirnov test was used to compare the empirical cumulative distribution function of sample data with the normal distribution. The analysis of Kruskal–Wallis Test, and χ2 test, as appropriate, were used to compare participants between the different combinations of cardiometabolic risk factors, respectively. Hazard ratios (HRs) and 95% confidential internal (CI) were calculated by using Cox proportional hazards models and the proportional hazard assumption were tested by weighted Schoenfeld residuals and *P*-values were greater than 0.05 for all outcomes. For all analyses, we adjusted for age, sex, BMI, alcohol consumption, smoking, educational level, employment status, urbanization (urban and rural), geographical region (east, central and western) and family history of CVD at baseline to assess accurately the relationship between individual and combined cardiometabolic disorders and the risk for CVD. Additionally, regarding that the potential impact of non-cardiovascular death as competing risk events rather than the censored, competing-risks regression based on Fine and Gray’s proportional sub-hazards model was used to evaluate this association [20, 21]. Then we redefined hypertension as the SBP ≥ 130 and/or DBP ≥ 80 according to the American Heart Association/ American College of Cardiology (AHA/ACC), and then assessed the association of the individual and combined cardiometabolic disorders with the risk for CVD and all-cause mortality. Besides, we used Poisson regression model to obtain CVD incidence rate and mortality rate for different combination of cardiometabolic risk factors adjusting for sex, age (liner and quadratic terms) and interactions of age at baseline with cardiometabolic risk factors.

The PAF for CVD and mortality attributable to hypertension, diabetes and high LDL-C separately was calculated using the formula [22]:

$${PAF}_i=P_i\times({HR}_i-1)/\lbrack P_i\times({HR}_i-1)+1\rbrack,$$

where ${P}_{i}$ is the actual prevalence of hypertension, diabetes and high LDL-C, and ${HR}_{i}$ is the adjusted hazard ratio of CVD and mortality associated with hypertension, diabetes or high LDL-C. We then computed the PAF for combined effects of cardiometabolic risk factors, which were considered as independent risk factors in this current study, by the following formula [23]:

$$PAF=1-\prod\nolimits_{i=1}^n(1-{PAF}_i),$$

where ${PAF}_{i}$ is the PAF of individual risk factors. We reported the 2·5th and 97·5th values as 95% CIs, which were computed via 1000 bootstrap resampling. Lastly, we evaluated the loss of CVD-free years and reductions in life expectancy related to different status of cardiometabolic disorders according to trapezium rule. Briefly, reductions in CVD-free years and life expectancy were estimated as the different of areas under any two survival curves compared based on multivariate adjusted Cox proportional models, with age as the timescale. The formula was [11]:

$$\mathrm{Loss}\;\mathrm{of}\;\mathrm{CVD}\;-\;\mathrm{free}\;\mathrm{years}\;=\int\nolimits_{\mathrm{baseline}\;\mathrm{age}}^{95}\;\left\{{\mathrm S}_{\mathrm{ref}}\left(\mathrm\mu\right)-{\mathrm S}_{\mathrm i}\left(\mathrm\mu\right)\right\}\mathrm{du},$$

where ${S}_{ref}\left(\mu \right)$ is the survival probability for participants without any of the three cardiometabolic disorders at age μ, and ${S}_{i}\left(\mu \right)$ is the survival probability at age μ for other status of cardiometabolic disorders (*i*).

All analyses were performed by using SAS version 9.4 (SAS Institute) and R version 4.0.3 (the R Foundation), and a two-sided *P* value < 0.05 was statistically significant.

## Results

### Characteristics of participants

At baseline, the mean age was 56.2 ± 13.1 years. Baseline characteristics based upon the numbers of cardiometabolic risk factors were demonstrated in Table 1. Of these 22,596 participants, most of them had a history of only one of hypertension, diabetes and high LDL-C (37.33%), 8.28% had two components for cardiometabolic factors, 6.06% had a history of diabetes, hypertension and high LDL-C. Compare with participants without cardiometabolic disorders, those with two or three cardiometabolic risk factors were older, were more likely to live in eastern urban areas and had a family history of CVD. Overall, BMI, FPG, and lipid levels increased markedly as the cardiometabolic health deteriorated, and mean SBP was as high as 152.7 mmHg in patients with all three cardiometabolic risk factors. Baseline characteristics of the sample population and the participants included in the final analysis are showed in Table S1.

**Table 1:** Characteristics of study participants by the number of cardiometabolic risk factors

Characteristic	None (*N* = 12,153)	One risk factor (*N* = 8436)	Two risk factors (*N* = 1870)	Three risk factors (*N* = 137)	*P* value
**Age (SD), years**	51.9 (12.2)	60.6 (12.5)	62.9 (11.8)	64.3 (11.8)	< .001
**Gender, %**					< .001
Male	5409 (44.5)	4154 (49.2)	832 (44.5)	61 (44.5)	
Female	6744 (55.5)	4282 (50.8)	1038 (55.5)	76 (55.5)	
**Educational level, %**					< .001
Elementary middle School or lower	5759 (47.4)	4642 (55.0)	1052 (56.3)	81 (59.1)	
High school or above	6394 (52.6)	3794 (45.0)	818 (43.7)	56 (40.9)	
**Residence, %**					< .001
Urban	5165 (42.5)	3913 (46.4)	931 (49.8)	75 (54.7)	
Rural	6988 (57.5)	4523 (53.6)	939 (50.2)	62 (45.3)	
**Region, %**					< .001
East	4801 (39.5)	3380 (40.1)	961 (51.4)	77 (56.2)	
Central	5097 (41.9)	3636 (43.1)	630 (33.7)	42 (30.7)	
West	2255 (18.6)	1420 (16.8)	279 (14.9)	18 (13.1)	
**Consumption of** **Alcohol, %**	3355 (27.6)	2406 (28.5)	488 (26.1)	40 (29.2)	0.156
**Smoking types, %**					< .001
Current	3093 (25.5)	2167 (25.7)	404 (21.6)	34 (24.8)	
Former	502 (4.1)	557 (6.6)	144 (7.7)	6 (4.4)	
Never	8558 (70.4)	5712 (67.7)	1322 (70.7)	97 (70.8)	
**SBP (SD), mm Hg**	119.7 (10.9)	145.1 (19.4)	149.2 (18.3)	152.7 (19.9)	< .001
**BMI, Kg/m**^**2**^	24.5 (3.5)	23.9 (3.3)	25.1 (3.6)	26.0 (3.5)	< .001
**Cholesterol (SD), mmol/L**					
Total	4.6 (0.8)	4.9 (1.0)	5.5 (1.3)	6.9 (0.8)	< .001
HDL-C	1.4 (0.3)	1.3 (0.3)	1.3 (0.3)	1.4 (0.4)	< .001
LDL-C	2.6 (0.7)	2.9 (0.8)	3.4 (1.1)	4.7 (1.1)	< .001
**FPG (SD), mmol/L**	5.1 (0.6)	5.7 (1.6)	7.5 (2.7)	8.7 (2.8)	< .001
**Family history of CVD, %**	1184 (9.7)	1574 (18.7)	477 (25.5)	39 (28.5)	< .001

Data are represented as mean (standard deviation) or number (%). *SBP* systolic blood pressure, *BMI* body mass index, *HDL-C* high-density lipoprotein cholesterol, *LDL-C* low density lipoprotein cholesterol, *FPG* fasting plasma glucose, *CVD* cardiovascular disease

### Associations of cardiometabolic disorders with CVD and all-cause mortality risk

**Fig. 1:**
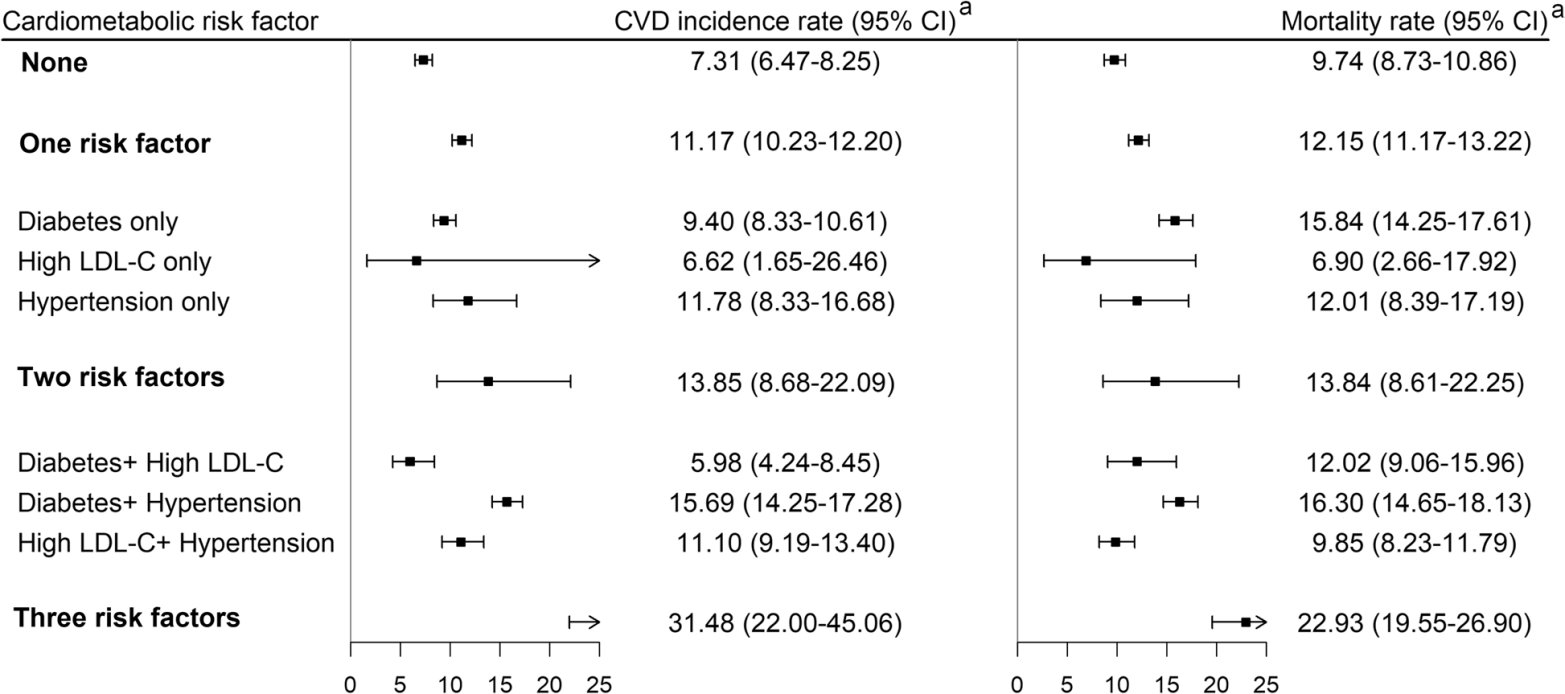
Sex- and age- adjusted CVD incidence and all-cause mortality rates by different combinations of cardiometabolic risk factors. CVD, cardiovascular disease; CI, confidential interval. ^a^, CVD incidence and mortality rate is per 1000 person-years

During the follow-up period, we detected 991 individuals with incident fatal or nonfatal CVD events (stroke, 596; CHD, 303; and other cardiovascular events, 92), the cumulative incidence of CVD and all-cause mortality were 9.48 and 10.83 per 1000 person-years. Table 2 showed the association between different combinations of cardiometabolic risk factors and the risk of CVD and all-cause mortality. Multivariable-adjusted model showed a significantly high risk of new-onset CVD among those with one, two or three cardiometabolic risk factors compared with participants without diabetes, hypertension, and high LDL-C, with the HR were 1.59 (95%CI 1.37–1.85), 1.82 (95%CI 1.49–2.24) and 2.97 (95%CI 1.85–4.75), respectively. Subjects with the combination of diabetes, hypertension, and high LDL-C had greater than twofold increased risk for CVD, CHD, stroke and all-cause mortality. For participants with only one cardiometabolic risk component, those with only hypertension showed significantly higher risk for stroke. However, we failed to detect an increased risk for CVD and its subcategories in participant with diabetes or high LDL-C only. Additionally, participants with diabetes only at baseline had a higher risk for all-cause mortality (HR = 1.79, 95%CI 1.36–2.37). Moreover, the HRs were 2.06 (95%CI 1.64–2.58) and 1.45 (95%CI 1.03–2.05) for total CVD in participants with diabetes plus hypertension and high LDL-C plus hypertension. Specifically, the risks of CHD incidence for participants with diabetes plus hypertension substantially increased through roughly 2.41 times compared with those without cardiometabolic risk factor, which was also higher than those with high LDL-C plus hypertension (HR = 2.06 (95%CI 1.17–3.63)). Moreover, for CVD risk, the conclusions were consistent after non-cardiovascular death was considered as competing risk events rather than the censored based on the Fine-Gray model (Table S2). After we redefined the hypertension according to the AHA/ACC, the risk of stroke increased further for participants with one or two cardiometabolic risk factors. In the reference group, the sex- and age- adjusted CVD incidence rate and mortality rate were 7.31 and 9.74 per 1000 person-years. By contrast, the age- and sex- adjusted CVD incidence and mortality rate were 31.48 (95%CI 22.00–45.06) and 22.93 (95%CI 19.55–26.90) per 1000 person-years among participants with diabetes, high LDL-C and hypertension (Fig. 1).

**Table 2:** HR for CVD and all-cause mortality associated with different combination of cardiometabolic risk factors

	**No. of participants**	**CVD**		**Stroke**		**CHD**		**All-cause mortality**	
		**Cases**	**HR (95%CI)**	**Cases**	**HR (95%CI)**	**Cases**	**HR (95%CI)**	**Cases**	**HR (95%CI)**
**The number of cardiometabolic risk factors**									
None	12,153	282	Reference	170	Reference	78	Reference	349	Reference
One	8436	533	1.59 (1.37–1.85)	328	1.65 (1.36–2.00)	160	1.63 (1.23–2.16)	593	1.38 (1.20–1.58)
Two	1870	157	1.82 (1.49–2.24)	88	1.67 (1.27–2.18)	57	2.30 (1.61–3.30)	164	1.66 (1.37–2.02)
Three	137	19	2.97 (1.85–4.75)	10	2.51 (1.31–4.78)	8	3.92 (1.86–8.23)	20	2.97 (1.88–4.69)
**The type of cardiometabolic risk factors**									
None	12,153	282	Reference	170	Reference	78	Reference	349	Reference
Diabetes only	830	36	1.31 (0.93–1.86)	20	1.22 (0.76–1.94)	14	1.83 (1.03–3.24)	58	1.79 (1.36–2.37)
High LDL-C only	632	15	0.82 (0.49–1.38)	8	0.70 (0.34–1.42)	7	1.52 (0.70–3.30)	16	0.76 (0.46–1.25)
Hypertension only	6974	482	1.68 (1.44–1.96)	300	1.78 (1.46–2.17)	139	1.62 (1.21–2.16)	519	1.37 (1.19–1.58)
Diabetes + High LDL-C	73	2	0.77 (0.19–3.08)	0	-	1	1.51 (0.21–10.92)	4	1.28 (0.48–3.43)
Diabetes + Hypertension	1236	116	2.06 (1.64–2.58)	64	1.90 (1.40–2.56)	41	2.41 (1.62–3.59)	121	1.92 (1.55–2.39)
High LDL-C + Hypertension	561	39	1.45 (1.03–2.05)	24	1.42 (0.92–2.20)	15	2.06 (1.17–3.63)	39	1.16 (0.83–1.62)
Diabetes + High LDL-C + hypertension	137	19	3.02 (1.88–4.83)	10	2.56 (1.34–4.89)	8	3.96 (1.89–8.33)	20	3.02 (1.92–4.77)

*CVD* cardiovascular disease, *CI* confidential interval, *CHD* coronary heart disease, *HR* hazard ratio, *LDL-C* low-density lipoprotein cholesterol

### Population attributable fraction and reductions in CVD-free years and life expectancy

The proportion of the total CVD attributable to diabetes and hypertension were 2.65% (95%CI 2.50–2.81) and 19.35% (95%CI 18.97–19.72), respectively (Table 3). We failed to detect a significant association between high LDL-C and the risk of CVD, therefore, we did not further calculate the PAF. The PAFs for all-cause mortality attributable to diabetes and hypertension were 5.08% (95%CI 4.80–5.37) and 10.85% (95%CI 10.61–11.08), respectively. Besides, we found that 21.48% of the CVD and 15.38% of all-cause mortality were attributable to the combined effect of diabetes and hypertension (Table 3).

**Table 3:** Population attributable fractions for CVD and all-cause mortality associated with cardiometabolic risk factors

Risk factor	Weight prevalence (%)	Outcomes	HR (95% CI)	PAF % (95%CI)
**Diabetes mellitus**	9.13 (8.61–9.69)	CVD	1.30 (1.10–1.54)	2.65 (2.50–2.81)
		Stroke	1.13 (0.90–1.41)	1.16 (1.10–1.23)
		CHD	1.59 (1.20–2.12)	5.15 (4.86–5.44)
		All-cause mortality	1.59 (1.36–1.85)	5.08 (4.80–5.37)
**Hypertension**	34.76 (33.92–35.60)	CVD	1.69 (1.47–1.94)	19.35 (18.97–19.72)
		Stroke	1.79 (1.50–2.15)	21.55 (21.13–21.95)
		CHD	1.56 (1.21–2.02)	16.29 (15.96–16.62)
		All-cause mortality	1.35 (1.19–1.53)	10.85 (10.61–11.08)
**High LDL-C**	5.40 (5.03–5.76)	CVD	0.94 (0.74–1.20)	-
		Stroke	0.82 (0.60–1.13)	-
		CHD	1.38 (0.95–2.02)	-
		All-cause mortality	0.93 (0.74–1.17)	-
**Combined effects**^**a**^	-	CVD	-	21.48 (21.09–21.90)
		Stroke		22.46 (22.04–22.87)
		CHD		20.60 (20.21–21.04)
		All-cause mortality		15.38 (15.05–15.74)

*CVD* cardiovascular disease, *HR* hazard ratio, *PAF* Population attributable fraction, *CI* confidential interval, *CHD* coronary heart disease, *LDL-C* low density lipoprotein cholesterol

^a^Combined PAF attributable to hypertension and diabetes

**Fig. 2:**
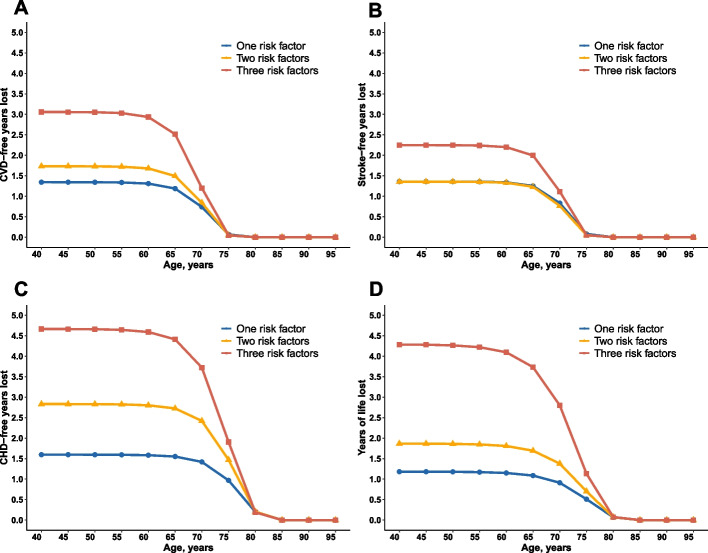
CVD-free years lost and years of life lost from 40 years of age onward in different numbers of cardiometabolic risk factors. CVD-free years lost and years of life lost were calculated in participants with one, two or three cardiometabolic disorders compared with participants without diabetes, hypertension and high HDL-C

We estimated that participants aged between 40 and 60 years old, with three cardiometabolic disorder together, would develop CVD on average 3.1 years earlier compared with those without any abnormalities of cardiometabolic factors at baseline (Fig. 2A). Subjects with one and two combinations of cardiometabolic risk factors had lost 1.3 and 1.7 CVD disease-free years, respectively. Corresponding reductions for CHD-free were 4.7 years among those had all three cardiometabolic risk factors, however, the reduction in stroke-free years was 2.2, which was slightly lower than the loss for CHD-free years (Figs. 2B and C). The loss of CVD disease-free years decreased with age for people who had cardiometabolic disorders after the age of 60. Besides, individuals over 75 years of age with identified cardiometabolic risk factors were substantially no CVD-free year loss. Estimated reductions in life expectancy in participants with all three cardiometabolic risk factors were 4.3 years at the index of 40 (Fig. 2D).

## Discussion

The current prospective study found that compared with participants without diabetes, hypertension or high LDL-C, those who had cardiometabolic multi-morbidities had a higher risk of incident CVD and all-cause mortality, and the cardiometabolic risk factors showed a multiplicative increased risk for CVD and all-cause mortality. Besides, The PAFs for CVD, CHD, stroke and all-cause mortality attributable to combined effect of diabetes and hypertension were 21.48%, 22.46%, 20.60% and 15.38%, respectively. Our results also suggested that both individual and combined cardiometabolic risk factors among participants aged between 40 and 60 years old are associated with significant reductions in CVD-free years and life expectancy compared participants without any of diabetes, hypertension or high LDL-C.

Cardiovascular disease are the leading cause of disability and death, and most of them are attributable to cardiometabolic risk factors, such as hypertension, diabetes, and high LDL-C [9, 10, 24, 25]. Recent results from the China Cardiometabolic Disease and Cancer Cohort (4C) study showed that approximately 37.42% of major cardiovascular events are attributable to the clustering of metabolic risk factors [10]. However, comprehensive control rate of hypertension, diabetes and dyslipidemia was low, a study based on Chinese participants, including 25,817 patients with diabetes, demonstrated that 72% of patients with type 2 diabetes have hypertension and/or dyslipidemia, and the integrated control rate is only 5.6% [26]. Chinese government proposed integration for hypertension, diabetes and dyslipidemia, which is in line with the principles of health economics and integrated management and control for cardiovascular disease. This study found that associations of cardiometabolic multi-morbidities with the risk of CVD incidence and all-cause mortality are multiplicative, which is of paramount importance to provide evidence for the primary prevention of CVD and comprehensive management for hypertension, diabetes and dyslipidemia, in China, and other countries.

While several studies have focus on the association of hypertension, diabetes and dyslipidemia with CVD risk separately [8, 9, 27], few have systematically clarified the relative association between combined cardiometabolic disorders with CVD, especially for Chinese people. 4C study, recruiting 193,846 adults aged over the age of 40, investigated the associations of individual and combined hypertension, diabetes and dyslipidemia with CVD risk among Chinese, and found that people with only hypertension or diabetes have a significantly increased risk of CVD compared with adults without hypertension, diabetes or dyslipidemia [10]. However, it failed to find an association of dyslipidemia only with the risk of CVD incidence, which was consistent with our study. One of the reasons is that the definition of dyslipidemia was based on abnormal TC, LDL-C, and HDL-C, and previous study showed inconsistent association between HDL-C and CVD risk [28–30], which may affect the estimation of the effect and overall test efficacy. Our findings also indicated hypertension is the largest cardiometabolic risk factor for total CVD and all-cause mortality, and accounting for approximately 19.35% and 10.85% of the PAF, respectively. A longitudinal cohort study enrolling 1,038,704 adults in China revealed that 3.3%, 1.73% and 0.3% all-cause mortality are due to one, two and three cardiometabolic multimorbidity [31]. Moreover, the latest research revealed that there are about 245 million adults in China have hypertension [4], indicating that CVD burden caused by hypertension remain serious, thus preventive interventions should be implemented to lower the prevalence of high SBP among Chinese overall in order to reduce the SBP-related CVD adverse health effect.

Our results noted that most combinations of cardiometabolic risk factors are associated with significant reductions in CVD-free years and life expectancy compared participants without diabetes, hypertension or high LDL-C among participants aged between 40 and 60 years old. Although several previous studies have investigated the association of cardiometabolic multi-morbidities with all-cause mortality and the associated reduction in life expectancy, these studies have basically focused on secondary prevention for CVD rather than primary prevention [31–33], and limited large-scale studies were conducted to explore the reduction in CVD-free years associated with individual and combined cardiometabolic disorders. Recent research noted that people with cardiometabolic multi-morbidity have significantly lower life expectancy and any combination of multi-morbidity was associated with the increased risk of all-cause mortality [33]. However, most of these studies focus on secondary prevention, and the definition of cardiometabolic multi-morbidity mainly includes stroke, myocardial infarction, diabetes. This present study, balancing the primary and secondary prevention of CVD, clarified that the relationship between different combination of cardiometabolic risk factor and CVD risk and the losses of CVD-free years caused by cardiometabolic risk factors. Recent researches have presented that healthy lifestyle can effectively reduce cardiometabolic diseases [34, 35], thus, popularizing health education and advocating a healthy lifestyle is a cost-effective approach to the primary prevention of CVD and the reduction of CVD burden caused by cardiometabolic risk factors, especially for people under 65 years of age.

### Strengths and limitations

There are several strengths in this study. Firstly, to our best knowledge, our study is the first to assess the PAF for CVD and its subcategories attributable to different combination of hypertension, diabetes and high LDL-C among Chinese people. Moreover, findings from our study emphasized the importance of an integrated management of cardiometabolic morbidities in order to improve the efficiency of prevention and control of CVD. However, we acknowledge some specific limitations. First, the relatively short follow-up duration limited the number of CVD events, so this study did not provide the loss of CVD-free years for all possible combinations of cardiometabolic disorders. In addition, we do not distinguish between ischemic and hemorrhagic strokes although the effects of cardiometabolic risk factors may vary among different types of strokes. Third, we were unable to further assess the association of individual and combined cardiometabolic risk factor with the risk of CVD incidence by different treatment status due to the limited sample size. Forth, it’s better to apply a more complex stratified multistage sampling method to have a more representative sample, namely, in the first stage, probability proportional to size should be used to obtain cities/counties stratified by urban and rural areas and economic level. Besides, information on the cardiometabolic risk factors may change over time, which could increase the possibility of misclassification bias during the follow-up and influence the accuracy of the estimates. Finally, although extensive covariates were adjusted in the analyses, residual and unmeasured confounders and intricate interaction remain exit, which may influence the precise effects of cardiometabolic risk factors on the risk of CVD incidence.

## Conclusions

In summary, our finding shows that cardiometabolic risk factors, including hypertension, diabetes and high LDL-C, were associated with multiplicative risk for CVD incidence and all-cause mortality, and a large proportion of CVD and mortality were attributable to cardiometabolic risk factors. The reduction in CVD-free years and life expectancy were substantially higher among people with combination of two and three cardiometabolic risk factors. This result highlights the importance of cardiometabolic multi-morbidities in the primary prevention of CVD and comprehensive management for hypertension, diabetes and abnormal lipids.

### Supplementary Information

**Additional file 1:** **Table S1.** Baseline characteristics of the sample population and the participants included in the analysis. **Table S2.** HR for CVD associated with different combination of cardiometabolic risk factors using competing risk model. **Table S3. **HR for CVD and all-cause mortality associated with different combination of cardiometabolic risk factors. **Figure S1.** Flow chart of study participants included and excluded in the study.

## Data Availability

The datasets used and/or analysed during the current study are available from the corresponding author on reasonable request.

## Abbreviations

CVD: Cardiovascular disease
PAF: Population attributable fraction
CI: Confidential interval
CHS: China Hypertension Survey
CHD: Coronary heart disease
SBP: Systolic blood pressure
FPG: Fasting plasma glucose
TC: Total cholesterol
TG: Triglycerides
LDL-C: Low-density lipoprotein cholesterol
HDL-C: High-density lipoprotein cholesterol
BMI: Body mass index
ICD-10: The International Classification of Diseases, 10th Revision
HR: Hazard ratio
4C: The China Cardiometabolic Disease and Cancer Cohort
