# Supplementary material for: Cardiovascular disease and all-cause mortality associated with individual and combined cardiometabolic risk factors
Source: BMC Public Health. 2023 Sep 5;23:1725. doi: 10.1186/s12889-023-16659-8 (PMC10478453; doi:10.1186/s12889-023-16659-8)
Supplement: Supplementary file 1 — Additional file 1: Table S1. Baseline characteristics of the sample population and the participants included in the analysis. Table S2. HR for CVD associated with different combination of cardiometabolic risk factors using competing risk model. Table S3. HR for CVD and all-cause mortality associated with different combination of cardiometabolic risk factors. Figure S1. Flow chart of study participants included and excluded in the study. [file 12889_2023_16659_MOESM1_ESM.docx]

**Supplementary materials**

**Table S1.** Baseline characteristics of the sample population and the participants included in the analysis.

**Table S2.** HR for CVD associated with different combination of cardiometabolic risk factors using competing risk model.

**Table S3.** HR for CVD and all-cause mortality associated with different combination of cardiometabolic risk factors.

**Figure S1.** Flow chart of study participants included and excluded in the study.

**Table S1. Baseline characteristics of the sample population and the participants included in the analysis.**

| **Characteristic** | **Incomplete data**  **(N=30036)** | **Complete data**  **(N=22660)** | ***P* value** | **Absolute standardized difference, %** |
| --- | --- | --- | --- | --- |
| **Age (SD), years** | 57.0 (13.3) | 56.2 (13.1) | <.001 | 6.6 |
| **Gender, %** |  |  | 0.839 | 0.2 |
| Male | 13872 (46.2) | 10456 (46.3) |  |  |
| Female | 16164 (53.8) | 12140 (53.7) |  |  |
| **Educational level, %** |  |  | <.001 | 4.0 |
| Elementary middle School or lower | 14733 (49.1) | 11534 (51.0) |  |  |
| High school or above | 15300 (50.9) | 11062 (49.0) |  |  |
| **Residence, %** |  |  | <.001 | 10.3 |
| Urban | 14943 (49.8) | 10084 (44.6) |  |  |
| Rural | 15093 (50.2) | 12512 (55.4) |  |  |
| **Region, %** |  |  | 0.014 |  |
| East | 12526 (41.7) | 9219 (40.8) |  | 1.8 |
| Central | 12122 (40.4) | 9405 (41.6) |  | 2.4 |
| West | 5388 (17.9) | 3972 (17.6) |  | 0.8 |
| **Consumption of Alcohol, %** | 8235 (27.4) | 6289 (27.8) | 0.295 | 0.9 |
| **Smoking types, %** |  |  | <.001 |  |
| Current | 7302 (24.3) | 5698 (25.2) |  | 2.1 |
| Former | 1930 (6.4) | 1209 (5.4) |  | 4.2 |
| Never | 20801 (69.3) | 15689 (69.4) |  | 0.2 |
| **SBP (SD), mm Hg** | 132.4 (20.4) | 131.9 (20.1) | 0.001 | 2.9 |
| **BMI, Kg/m^2^** | 24.6 (3.5) | 24.5 (3.5) | <.001 | 3.2 |
| **Cholesterol (SD), mmol/L** |  |  |  |  |
| Total | 4.8 (1.0) | 4.8 (1.0) | 0.097 | 1.5 |
| HDL-C | 1.4 (0.3) | 1.4 (0.3) | 0.445 | 0.7 |
| LDL-C | 2.8 (0.8) | 2.8 (0.8) | 0.130 | 1.3 |
| **FPG (SD), mmol/L** | 5.6 (1.6) | 5.6 (1.5) | <.001 | 4.4 |
| **Family history of CVD, %** | 4663 (15.5) | 3274 (14.5) | 0.001 | 2.9 |

Data are represented as mean (standard deviation) or number (%). SBP, systolic blood pressure; BMI, body mass index; HDL-C, high-density lipoprotein cholesterol; LDL-C, low density lipoprotein cholesterol; FPG, fasting plasma glucose; CVD, cardiovascular disease.

**Table S2. HR for CVD associated with different combination of cardiometabolic risk factors using competing risk model.**

|  | **No. of participants** | **CVD** | | **Stroke** | | **CHD** | |
| --- | --- | --- | --- | --- | --- | --- | --- |
|  |  | **Cases** | **HR (95%CI)** | **Cases** | **HR (95%CI)** | **Cases** | **HR (95%CI)** |
| **The number of cardiometabolic risk factors** | | | | | | | |
| None | 12153 | 282 | Reference | 170 | Reference | 78 | Reference |
| One | 8436 | 533 | 1.59 (1.36-1.85) | 328 | 1.59(1.36,1.85) | 160 | 1.59(1.36,1.85) |
| Two | 1870 | 157 | 1.84(1.49,2.27) | 88 | 1.67 (1.27-2.18) | 57 | 1.84(1.49,2.27) |
| Three | 137 | 19 | 3.02(1.83,4.97) | 10 | 3.02(1.83,4.97) | 8 | 3.02(1.83,4.97) |
| **The type of cardiometabolic risk factors** | | | | | | | |
| None | 12153 | 282 | Reference | 170 | Reference | 78 | Reference |
| Diabetes only | 830 | 36 | 1.31(0.92-1.86) | 20 | 1.21(0.76-1.93) | 14 | 1.83(1.03-3.26) |
| High LDL-C only | 632 | 15 | 0.82(0.49-1.39) | 8 | 0.70(0.34-1.42) | 7 | 1.52(0.70-3.31) |
| Hypertension only | 6974 | 482 | 1.68(1.43-1.97) | 300 | 1.78(1.45-2.19) | 139 | 1.61(1.19-2.18) |
| Diabetes+ High LDL-C | 73 | 2 | 0.76(0.19-3.03) | 0 | - | 1 | 1.49(0.21-10.47) |
| Diabetes+ Hypertension | 1236 | 116 | 2.08(1.64-2.63) | 64 | 1.91(1.40-2.60) | 41 | 2.42(1.61-3.64) |
| High LDL-C + Hypertension | 561 | 39 | 1.46(1.04-2.05) | 24 | 1.44(0.93-2.23) | 15 | 2.08(1.19-3.63) |
| Diabetes+ High LDL-C +hypertension | 137 | 19 | 3.07(1.87-5.06) | 10 | 2.59(1.32-5.07) | 8 | 3.99(1.87-8.51) |

CVD, cardiovascular disease; CI, confidential interval; CHD, coronary heart disease; HR, hazard ratio; LDL-C, low-density lipoprotein cholesterol.

**Table S3.** **HR for CVD and all-cause mortality associated with different combination of cardiometabolic risk factors*****.**

|  | **No. of participants** | **CVD** | | **Stroke** | | **CHD** | | **All-cause mortality** | |
| --- | --- | --- | --- | --- | --- | --- | --- | --- | --- |
|  |  | **Cases** | **HR (95%CI)** | **Cases** | **HR (95%CI)** | **Cases** | **HR (95%CI)** | **Cases** | **HR (95%CI)** |
| **The number of cardiometabolic risk factors** | | | | | | | |  |  |
| None | 8151 | 163 | Reference | 90 | Reference | 50 | Reference | 208 | Reference |
| One | 11860 | 631 | 1.58 (1.32-1.89) | 397 | 1.83 (1.45-2.33) | 179 | 1.36 (0.99-1.88) | 702 | 1.36 (1.16-1.59) |
| Two | 2407 | 177 | 1.81 (1.45-2.26) | 99 | 1.82 (1.35-2.45) | 66 | 2.08 (1.42-3.06) | 194 | 1.69 (1.38-2.08) |
| Three | 178 | 20 | 2.67 (1.67-4.28) | 10 | 2.37 (1.22-4.58) | 8 | 3.05 (1.42-6.51) | 22 | 2.69 (1.73-4.20) |
| **The type of cardiometabolic risk factors** | | | | | | | |  |  |
| None | 8151 | 163 | Reference | 90 | Reference | 50 | Reference | 208 | Reference |
| Diabetes only | 488 | 21 | 1.39 (0.88-2.19) | 12 | 1.44 (0.79-2.63) | 8 | 1.72 (0.81-3.64) | 32 | 1.74 (1.20-2.53) |
| High LDL-C only | 396 | 9 | 0.87 (0.44-1.67) | 5 | 0.83 (0.34-2.04) | 4 | 1.35 (0.49-3.76) | 10 | 0.85 (0.45-1.60) |
| Hypertension only | 10976 | 601 | 1.61 (1.35-1.93) | 380 | 1.90 (1.49-2.41) | 167 | 1.35 (0.97-1.87) | 660 | 1.35 (1.15-1.58) |
| Diabetes+ High LDL-C | 32 | 1 | 0.89 (0.13-6.39) | 0 | - | 1 | 3.27 (0.45-23.81) | 2 | 1.46 (0.36-5.90) |
| Diabetes+ Hypertension | 1578 | 131 | 2.02 (1.59-2.57) | 72 | 2.05 (1.48-2.83) | 47 | 2.15 (1.42-3.25) | 147 | 2.00 (1.61-2.49) |
| High LDL-C + Hypertension | 797 | 45 | 1.39 (1.00-1.95) | 27 | 1.47 (0.95-2.28) | 18 | 1.84 (1.06-3.20) | 45 | 1.10 (0.79-1.52) |
| Diabetes+ High LDL-C +hypertension | 178 | 20 | 2.70 (1.69-4.32) | 10 | 2.39 (1.23-4.62) | 8 | 3.08 (1.44-6.58) | 22 | 2.73 (1.75-4.23) |

CVD, cardiovascular disease; CI, confidential interval; CHD, coronary heart disease; HR, hazard ratio; LDL-C, low-density lipoprotein cholesterol.

* Cardiometabolic risk factors included diabetes, hypertension and high LDL-C. Hypertension was defined as systolic blood pressure ≥ 130mmHg, and /or diastolic blood pressure ≥ 80 mmHg, and/or self-reported physician-diagnosed hypertension, and/or medication use for antihypertensive at baseline.


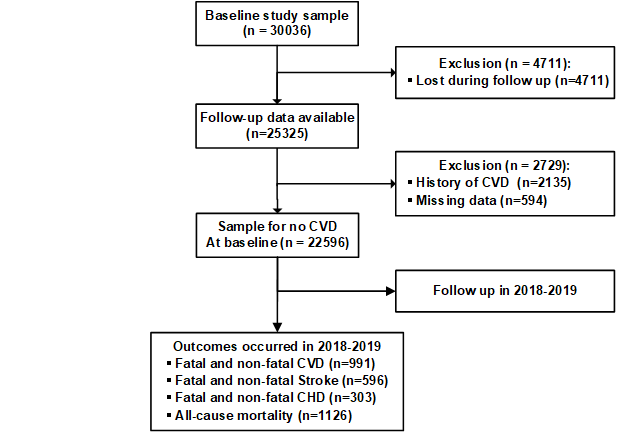


**Figure S1. Flow chart of study participants included and excluded in the study.** (CVD, cardiovascular disease; CHD, coronary heart disease)
